# Integrative genomic and functional characterization of ADAMTS3 reveals its inflammatory regulation via NF‐κB and STAT3 pathways in osteosarcoma

**DOI:** 10.1002/ccs3.70072

**Published:** 2026-05-03

**Authors:** Ehed Muhammed Aymaz, Meltem Alper, Feyza Nur Sav, Tuğşen Aydemir, Feray Köçkar

**Affiliations:** ^1^ İstinye University Graduate Education Institute Molecular Oncology Istanbul Turkey; ^2^ Department of Translational Oncology Dokuz Eylul University Institute of Oncology Izmir Turkey; ^3^ Faculty of Science and Literature Department of Molecular Biology and Genetics Balikesir University Balikesir Turkey; ^4^ Cell Therapy Department Moffitt Cancer Center and Research Institute Tampa Florida USA

**Keywords:** ADAMTS‐3, extracellular matrix remodeling, NF‐κB and STAT3 transcription factors, osteosarcoma, tumor necrosis factor‐alpha (TNF‐α) signaling pathway

## Abstract

Osteosarcoma (OS) is an aggressive bone malignancy characterized by genomic instability and extensive extracellular matrix (ECM) remodeling. Members of the *ADAMTS family* are matrix‐associated proteases implicated in tumorigenesis; however, their roles in OS remain poorly defined. This study provides a comprehensive genomic, transcriptomic, and functional analysis of the ADAMTSs in OS, with particular focus on ADAMTS‐3. Copy number alterations and mRNA expressions of ADAMTS genes were analyzed using the TCGA datasets. Gene set enrichment analysis and co‐expression analyses identified biological processes associated with ADAMTS‐3. Mechanistic studies investigated tumor necrosis factor‐alpha (TNF‐α) regulation of ADAMTS‐3 in OS cells. Genomic profiling revealed frequent amplification and high mRNA expression of ADAMTS4, ADAMTS12, ADAMTS16, and ADAMTS17, indicating potential oncogenic activity. ADAMTS‐3 was markedly overexpressed in OS tissues and cell lines, showing strong positive correlations with inflammatory (IL6, STAT3, NF‐κB) and matrix‐remodeling (MMP2, MMP9) genes. Functional enrichment indicated that ADAMTS‐3 is associated with ECM organization, immune response regulation, and epithelial‐mesenchymal transition. Mechanistically, TNF‐α induced ADAMTS‐3 transcription via activation of MEK, PI3K, JNK, and NF‐κB pathways, with STAT3 and NF‐κB by enhancing promoter activity. These findings identify ADAMTS‐3 as an inflammation‐responsive gene that links inflammatory signaling to ECM remodeling and tumor invasiveness in OS, representing a potential molecular bridge.

## Graphical abstract

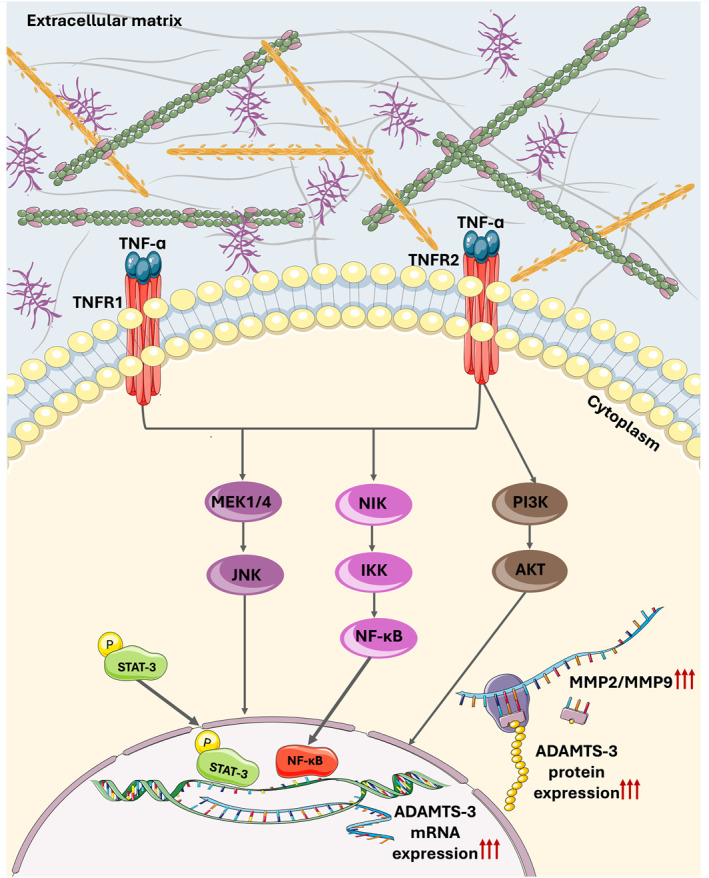

## 1 INTRODUCTION

Osteosarcoma (OS) is a rare but the most common primary malignant bone tumor affecting children and adolescents. Despite progress in multimodal treatments that combine surgery and chemotherapy, survival outcomes for patients with metastatic or relapsed disease remain unsatisfactory. Increasing evidence indicates that chronic inflammation within the tumor microenvironment is a key factor influencing OS initiation and progression. OS tissues often exhibit active immune infiltration, accompanied by elevated circulating levels of pro‐inflammatory cytokines such as tumor necrosis factor‐alpha (TNF‐α). These inflammatory mediators are thought to shape the tumor microenvironment, promoting proliferation, invasion, and resistance to therapy. However, the molecular mechanisms through which inflammatory signaling contributes to OS pathogenesis and metastatic dissemination are still poorly defined.^1^, ^2^, ^3^, ^4^, ^5^.

The inflammatory tumor microenvironment facilitates tumorigenesis through multiple mechanisms, including promoting angiogenesis, enhancing metastatic potential, suppressing antitumor immune responses, and altering cellular sensitivity to hormones and chemotherapeutic agents.^6^, ^7^ Among inflammatory mediators, cytokines such as TNF‐α and interleukins are known to regulate key transcriptional programs in cancer cells and surrounding stromal tissues, contributing to extracellular matrix (ECM) remodeling, invasion, and metastasis.^8^, ^9^

ADAMTS (ADAM metallopeptidase with thrombospondin Motifs) genes are highly relevant to ECM dynamics and inflammation‐driven tumor progression. ADAMTS proteins have the capacity to degrade or interact with various extracellular matrix (ECM) components and regulatory molecules, thereby influencing critical cellular processes, including adhesion, migration, proliferation, and angiogenesis. Alterations in ADAMTS gene expression—including overexpression, mutations, or epigenetic silencing—have been reported across multiple tumor types, suggesting a significant role for these metalloproteinases in cancer development.^10^, ^11^, ^12^

ADAMTS‐2, ‐3, and ‐14 are classified as procollagen N‐proteinases, owing to their ability to cleave the N‐terminal propeptide from fibrillar procollagen molecules.^13^ While ADAMTS‐2 targets multiple collagen types (I, II, III, and V), ADAMTS‐3 is predominantly responsible for processing type II collagen.^14^ ADAMTS‐3 is located on chromosome 4q13.3, comprises 23 exons, and encodes a 135 kDa protein.^15^ Compared to ADAMTS‐2, ADAMTS‐3 is more abundantly expressed in cartilage, where it functions as a key regulator of collagen II maturation. Its expression has also been detected in other tissues, such as the placenta, kidney, heart, and skin.^16^ Notably, elevated ADAMTS‐3 expression has been observed in pathological conditions such as myocardial infarction, osteoarthritis, and breast cancer, suggesting a possible role in tissue remodeling under both physiological and disease states.^16^

Recent studies have begun to explore the regulation of ADAMTS family members by inflammatory cytokines. For instance, TNF‐α was shown to upregulate ADAMTS‐1, ‐6, and ‐9 in ocular cell lines,^17^ while IL‐1β and Oncostatin‐M enhanced the expression of ADAMTS‐4 and ‐5 in cartilage cells.^18^ Similarly, TNF‐α and IL‐1β synergistically induce ADAMTS‐9 expression in human chondrocytes,^19^ although the downregulation of specific ADAMTS members, such as ADAMTS‐1, in pre‐adipocytes under TNF‐α treatment has also been reported. These findings underscore the cell‐type and context‐specific nature of cytokine‐mediated ADAMTS regulation.^19^

Despite growing interest in the role of ADAMTS proteases in inflammation and cancer, the transcriptional regulation of ADAMTS‐3, particularly in the context of TNF‐α signaling, remains poorly understood. NF‐κB and STAT3 transcription factors, activated downstream of cytokine receptors, are central mediators of inflammatory gene expression. Both NF‐κB and STAT3 have been implicated in tumor proliferation, survival, and immune evasion, and are frequently dysregulated in various malignancies, including OS.^20^, ^21^, ^22^

In this study, initially, we bioinformatically analyzed genomic and transcriptomic alterations of ADAMTS family members in OS. Further, We Investigated the Expression, Co‐expression, Functional Enrichment, and Clinical Associations of ADAMTS‐3 in OS. We aimed to study the regulatory effect of TNF‐α on ADAMTS‐3 at the mRNA, protein, and transcriptional activity levels using the Saos‐2 OS cell line as a model. We also evaluated the contribution of specific signaling pathways, including NF‐κB, STAT, MEK, JNK, and PI3K, to this regulatory process using pharmacological inhibitors and transcription factor binding assays. Our findings provide novel mechanistic insights into the inflammatory regulation of ADAMTS‐3 in OS, highlighting its potential role in the tumor microenvironment.

## 2 MATERIALS AND METHODS

### 2.1 Cell lines and cytokine treatment

Saos‐2 OS cell line were kindly provided by Dr. Kenneth Wann (Cardiff, School of Biosciences, Cardiff UK). Cells were tested for *mycoplasma* contamination. Saos‐2 OS cells were cultured in DMEM (Sigma) with 10% heat‐inactivated FCS and L‐glutamine (2 mM) (Invitrogen) under standard conditions (37°C, 5% CO_2_). Before stimulation, cells were serum‐deprived using 0.1% BSA and then treated with TNF‐α (10 ng/mL) up to 48 h.

### 2.2 Cell proliferation assay

MTT (0.5 mg/mL) was applied to both control and TNF‐α treated Saos‐2 cells in 96‐well plates (5 × 10^4 cells/well) and incubated for 4 h at 37°C in an incubator containing 5% CO_2_.^23^ Formazan crystals were solubilized in isopropanol with 0.004 M HCl, and absorbance was measured at 550 nm.^23^

### 2.3 RNA extraction and qRT‐PCR

Total RNA was extracted from Saos‐2 cells using GeneJET RNA Purification Kit (Thermo). Nanodrop was used to quantify RNA concentrations. cDNA was synthesized from 1 μg of total RNA using Reverse Transcriptase (200 U), oligo (dT) primer, and Ribonuclease inhibitor (40 U) at 42°C for 1 h. Real‐time PCR was conducted using 1 μL of cDNA from both control and cytokine‐treated groups, 5 μL of SYBR® Green PCR Master Mix, ADAMTS‐3‐specific forward and reverse primers (Table 1) in a 10 μL final volume using Light Cycler 485 (Roche Diagnostic). Cycling conditions were 95°C for 10 min, (95°C for 30 s, 55°C for 30 s, 72°C for 30 s) × 35 cycles and 72°C for 1 min for final extension. Samples were studied at least in triplicate. The Human β‐2 Microglobulin gene was used for normalization. The relative change in ADAMTS‐3 gene expression was evaluated using the Livak method.^24^

**TABLE 1:** Primer sequences for expression analysis of ADAMTS‐3 by PCR amplification.

Hβ‐2 sense	5′ TTTCTGGCCTGGAGGCTATC 3′
Hβ‐2 antisense	5′ CATGTCTCGATCCCACTTAACT 3′
ADAMTS‐3 sense	5′ GGAACACTGCACCAACCT 3′
ADAMTS‐3 antisense	5″ TGTCTCCCAAACATGGTTCA 3″

### 2.4 Western blotting

Protein extracts were prepared with RIPA buffer (10 mM Tris‐HCl, pH 8, 140 mM NaCl, 1 mM EDTA, 0.1% SDS, 1 mM EGTA, 1% Triton X‐100, 0.01% sodium deoxycholate) including protease inhibitor cocktail. Protein concentration was measured fluorometrically using Qubit. Equal amounts (50 μg) of protein samples were loaded on SDS‐PAGE. The wet transfer was conducted to a PVDF membrane overnight at +4°C. After blocking, the membrane was treated with ADAMTS‐3 antibody (1:500, y058016‐Abm) at +4°C overnight and the β‐Actin antibody (1:1000, Ab8227‐Abcam) for one hour at room temperature, then it was treated with the secondary antibody (1:5000, goat, anti‐rabbit)(Ab97069‐Abcam) for one hour at room temperature. Protein bands were detected by Fusion FX Vilber Lourmat using ECL Substrate (Thermo, Pierce). Image J was used for densitometric analysis.^25^

### 2.5 Immunofluorescence analysis

Saos‐2 cells were cultivated on coverslips with TNF‐α (500 U/mL) treatment. The analyses were carried out as described previously.^25^ Saos‐2 cells were incubated with ADAMTS‐3 (Abcam, Ab45037) antibody (2 μg/mL) overnight at 4°C. Secondary antibody was introduced into samples for 1 h at room temperature using Alexa Fluor 488 (anti‐rabbit, Invitrogen). An Olympus BX51 microscope with an ED200 fluorescence attachment was used for Fluorescence imaging. The photographs were taken with the Olympus DP72. ImageJ software was used to quantify the relative ADAMTS‐3 protein expression.

### 2.6 Inhibitor treatments

Saos‐2 cells were pre‐treated with pharmacological inhibitors for 30 min and then treated with TNF‐α (10 ng/mL) for 6 h. PD98059 (10 μM, Cell Signaling, 9900S) was used for MEK inhibition, Wortmannin (1 μM, Cell Signaling, 99515) was used for PI3K inhibition, SP600125 (20 μM, Santa Cruz, sc‐200635) was used for JNK inhibition, Bay 11‐7082 (5 μM, Santa Cruz, sc‐200615) was used for NFκB inhibition.^26^

### 2.7 Luciferase reporter assays

Four different truncated ADAMTS‐3 promoter constructs (1 μg) that were previously cloned into the pMetLuc Reporter vector and the pSeap2‐Control vector (0.5 μg) were used for transient transfection assays as described before.^27^ TNF‐α (10 ng/mL) was applied to Saos‐2 cells 16 h after transfection. Secreted luciferase and SEAP activities were measured after 48 and 72 h of stimulation using the Ready‐To‐Glow™Secreted Luciferase Reporter Systems (Clontech) and Fluoroskan Ascent FL Luminometer (Thermo Electron Co.). pMetLuc Control and pMetLuc reporter vectors were used as positive and negative controls to measure transfection efficiency and background activity. The data were normalized as the ratio of Luciferase to SEAP. Transfection studies were carried out at least three times.

### 2.8 EMSA (electrophoretic mobility shift assay)

Potential transcription factor binding motifs in the ADAMTS3 promoter region were predicted using the MatInspector tool (Genomatix).^28^ Probes [‐131/‐103], [‐324/‐295], [‐838/‐812], [‐973/‐937], [‐1084/‐1040], [‐1225/‐1205], [‐1278/‐1259] were used to analyze functional binding of STAT‐3 and/or NFκB with nuclear extracts from Saos‐2 cells treated with/without TNF‐α in EMSA. Biotinylated *ADAMTS‐3* promoter and unlabeled consensus STAT‐3 or NFκB oligonucleotides were used for competition assays. EMSA was performed as described previously using the LightShift Chemiluminescent EMSA Kit (Thermo Scientific). The biotin‐labeled DNA‐protein complex was visualized on a UVP imaging system using the ECL Substrate (Thermo, Pierce).^27^

### 2.9 Statistical analysis

MiniTab (One‐Way ANOVA) was used for statistical analysis. *p* ≤ 0.05 was considered statistically significant.

### 2.10 Bioinformatic analysis

The analyses were performed using the cBioPortal for Cancer Genomics platform (accessed: August 2025). The OS (TARGET GDC, 2025) dataset was selected as the study cohort (total of 159 samples). All visualisations were obtained from the portal's OncoPrint and Expression Heatmap panels. Copy number alterations (CNA) were selected from the “Genomic Profiles” section under the “Putative copy‐number alterations from GISTIC” profile. Normalised continuous CNA values were processed within cBioPortal using the Genomic Identification of Significant Targets in Cancer (GISTIC 2.0) algorithm.^29^ The case set was restricted to “Samples with CNA data (81).” Event types were reported according to GISTIC calls; amplifications were shown in red, “no change” events in grey. Event frequencies per gene were automatically calculated by the portal and presented as percentages on OncoPrint. The “mRNA Expression TPM z‐scores” profile was selected in the same study, and the threshold z‐score was defined as ≥2.0. Expression levels of ADAMTS‐3 across various cancer types were retrieved and analyzed from the Broad Institute's FireBrowse resource (http://firebrowse.org/). The boxplots produced showed the expression level of the target gene, with red bars representing tumor samples and blue bars representing normal samples.

The correlation statistic used is Spearman's, and the sample size is limited to *n* = 88 tumors with data available for this profile. The relationships between ADAMTS‐3 and IL6, IL1B, TNF, NFKB1, RELA, STAT3, PTGS2, MMP9, and MMP2 were examined individually, and the Spearman coefficient (ρ), *p*‐value, and Benjamini–Hochberg (FDR) *q*‐value reported by the portal were recorded (https://pubmed.ncbi.nlm.nih.gov/22588877/). Analyses were performed in the R (v4.5.x) environment. Gene expression data were obtained from the raw file of the NCBI GEO: GSE28424 study corresponding to the Illumina HumanHT‐12 v4 platform (GPL13376) (“GSE28424_non_normalized.txt”, AVG_Signal). Only OS cell line samples (*n* ≈ 19) were used; normal bone samples were excluded. Intensity values were subjected to log_2_(x+1) transformation, and quantile normalisation was applied using limma::normaliseBetweenArrays (method = “quantile”). In the resulting gene × sample matrix, the expression of each gene was calculated using Spearman's correlation coefficient (ρ) with ADAMTS‐3 levels across OS samples, and a pre‐ranked list was created in descending order based on the coefficients; ADAMTS‐3 was excluded from the ranking to prevent self‐enrichment. This list was pre‐ranked using clusterProfiler::GSEA. The Hallmark (H) and GO: Biological Process (GO: BP) collections were analyzed (cluster size limits 10–500 genes). For visualization, a scatter plot was generated using enrichplot/ggplot2; the *x*‐axis shows GeneRatio (core enriched gene/cluster size), color indicates FDR, and bubble size represents cluster size. The figure presents the top 15 most significant terms from the GO: BP results.

## 3 RESULTS

### 3.1 Genomic and transcriptomic landscape of the ADAMTS family in OS

**FIGURE 1:**
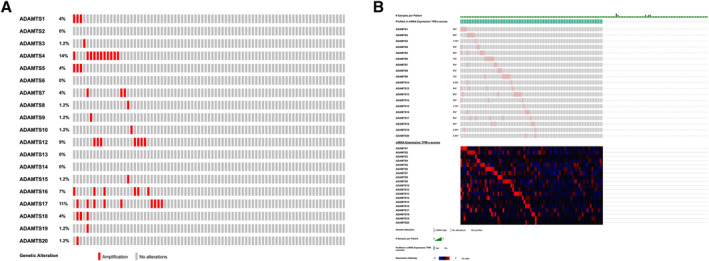
Genomic and transcriptomic alterations of the ADAMTS family in osteosarcoma. (A) Genomic alterations visualized using cBioPortal. (B) Transcriptomic alterations (mRNA expression, z‐score ≥2.0) of ADAMTS family genes in the same cohort.

Studies indicate that ADAMTS family members become dysregulated in cancer and play different roles in tumor progression. There is very limited information about the ADAMTS family in osteosarcomas. Meanwhile, somatic mutations in ADAMTS genes have been shown in different studies to be associated with chemotherapy sensitivity and patient survival.^30^, ^31^ ADAMTS mutations, CNA, and expression profiles were analyzed using CBioPortal from the TCGA (provisional) cohort. CNA (CNAs; *n* = 81) were evaluated for ADAMTS1–20. Amplification was observed at a very low frequency (1%; each ≈1/81) in ADAMTS3, ADAMTS8, ADAMTS9, ADAMTS10, ADAMTS15, ADAMTS19, and ADAMTS20. The amplification frequency was highest in ADAMTS4 at 14% (≈11/81) and ADAMTS17 at 11% (≈9/81); ADAMTS12 was intermediate at 9% (≈7/81) and ADAMTS16 at 7% (≈6/81). The ratio was calculated to be approximately 4% (3/81 each) for ADAMTS1, ADAMTS5, and ADAMTS18. No change in copy number was detected for ADAMTS2, ADAMTS6, ADAMTS13, and ADAMTS14 (0%). Amplifications were notably observed together in the same samples for ADAMTS4, ADAMTS16, and ADAMTS17 and were concentrated in specific patient subgroups (Figure 1A).

mRNA levels (TPM z‐score; *n* = 159) in the same cohort were examined using a z‐score threshold of ≥2.0 on OncoPrint. ADAMTS3 expression was mainly observed in the intermediate range. Significant heterogeneity was observed across the ADAMTS family in terms of expression, with high mRNA expression in ADAMTS4, ADAMTS12, ADAMTS16, and ADAMTS17 forming a typical pattern in patient tissues. In contrast, high expression cases were observed less frequently in ADAMTS2, ADAMTS6, ADAMTS13, and ADAMTS14. Although mRNA levels were occasionally elevated in the ADAMTS8, ADAMTS10, ADAMTS18, ADAMTS19, and ADAMTS20 genes, these increases were not consistent across the large patient group (Figure 1B). The heatmap also supports threshold‐based findings and details inter‐sample variability. The ADAMTS3 signal clustered around medium z‐scores and showed limited increases in only a small number of samples. High z‐score clusters in the form of broad red bands were evident in ADAMTS4, ADAMTS12, ADAMTS16, and ADAMTS17, while ADAMTS2, ADAMTS6, ADAMTS13, and ADAMTS14 were predominantly distributed in blue tones. Integratively, the partial overlap of amplification with high mRNA levels in ADAMTS4, ADAMTS16, and ADAMTS17 is consistent with a dose effect (Figure 1B).

### 3.2 Expression, co‐expression, functional enrichment, and clinical associations of ADAMTS3 in OS

**FIGURE 2:**
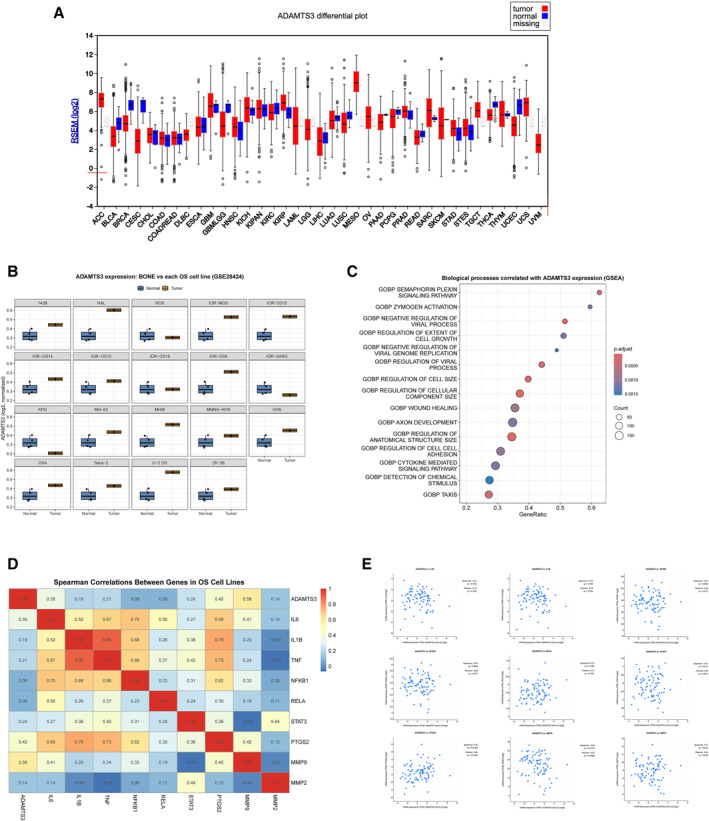
Expression, co‐expression, functional enrichment and clinical associations of ADAMTS‐3 in osteosarcoma. (A) Differential expression of ADAMTS3 across cancer types, comparing tumor (red) and normal (blue) tissues. (B) ADAMTS3 expression in osteosarcoma cell lines versus normal bone samples (GSE28424). (C) GSEA showing biological processes associated with ADAMTS3 expression; dot size indicates gene count and color represents adjusted *p*‐value. (D) Spearman correlation heatmap between ADAMTS3 and inflammation related genes in OS cell lines. (E) Scatter plots showing correlations between ADAMTS3 and inflammation related genes across samples.

The expression profile of ADAMTS‐3 in tumor and normal tissues was examined in TCGA cohorts using the Broad GDAC FireBrowse platform. Box‐plot visualisations revealed differences between primary tumor samples (red) and normal tissue samples (blue). In cohorts lacking normal tissue data, only tumor samples were evaluated. Overall, ADAMTS‐3 expression showed heterogeneous distribution across cancer types, with some tumor types exhibiting significant differences compared to normal tissues. In OS cohorts, ADAMTS‐3 expression was notably increased compared with normal tissue. This finding suggests that ADAMTS‐3 may play a specific role in OS biology (Figure 2A).

Comparative analyses in OS cell lines and normal osteoblasts revealed a marked increase in ADAMTS‐3 expression. Multi‐panel box plots revealed that ADAMTS‐3 levels were elevated in nearly all OS cell lines compared to normal osteoblasts. Although not statistically tested, this increase was consistently observed, suggesting that ADAMTS‐3 is generally overexpressed in OS cells (Figure 2B).

GSEA results obtained using OS cell lines revealed biological processes associated with ADAMTS‐3 expression. The analyses showed that genes co‐expressed with ADAMTS‐3 exhibited significant enrichment in GO: Biological Process (GO: BP) categories, particularly extracellular matrix organisation, cell adhesion, immune response regulation, and cellular proliferation. Furthermore, according to the Hallmark collection, gene clusters related to inflammatory response and epithelial‐mesenchymal transition also showed a positive correlation with ADAMTS‐3. These findings suggest that ADAMTS‐3 may be linked to both matrix remodeling and immune/inflammatory processes in OS biology (Figure 2C).

The remodeling of the extracellular matrix, invasion, and immune response mechanisms largely shapes the biology of OS. Although the ADAMTS family plays critical roles in these processes, knowledge regarding how ADAMTS‐3 is regulated in OS and which genes it interacts with functionally is limited. Therefore, elucidating the expression patterns of ADAMTS‐3 in conjunction with other genes is crucial for understanding the potential biological networks and molecular mechanisms that may influence tumor progression.

Using the GSE28424 set in the GEO database, co‐expression relationships between ADAMTS3 and other genes were evaluated in 19 OS cell lines. Normal bone samples were excluded, and only tumor cell lines were included in the analysis. Spearman correlation analyses revealed that ADAMTS3 showed significant positive correlations with inflammation‐related genes (IL6, IL1B, TNF, NFKB1, RELA, STAT3, and PTGS2) and matrix metalloproteinase genes (MMP2 and MMP9). The heat map confirmed that these genes exhibited similar clustering patterns to those of ADAMTS‐3. This suggests that ADAMTS‐3 may be strongly linked to genes associated with both the inflammatory response and extracellular matrix remodeling in OS cell lines (Figure 2D).

Scatter plots also confirm these findings. The clustering of points in the upper‐right region in ADAMTS‐3–MMP2/MMP9 pairings indicates that genes associated with invasion also show a parallel increase in lines with elevated ADAMTS‐3 levels. Positive correlations between ADAMTS‐3–IL6/STAT3/NFKB1/RELA suggest that ADAMTS‐3 may be regulated in both the IL‐6/JAK–STAT3 and NF‐κB axes. Furthermore, the positive trend observed in the ADAMTS‐3–PTGS2 relationship suggests that inflammation in the tumor microenvironment interacts with ADAMTS‐3 expression through COX‐2, potentially forming a bridge between matrix remodeling and the immune response (Figure 2E).

### 3.3 TNF‐α contributes to Saos‐2 cell proliferation

**FIGURE 3:**
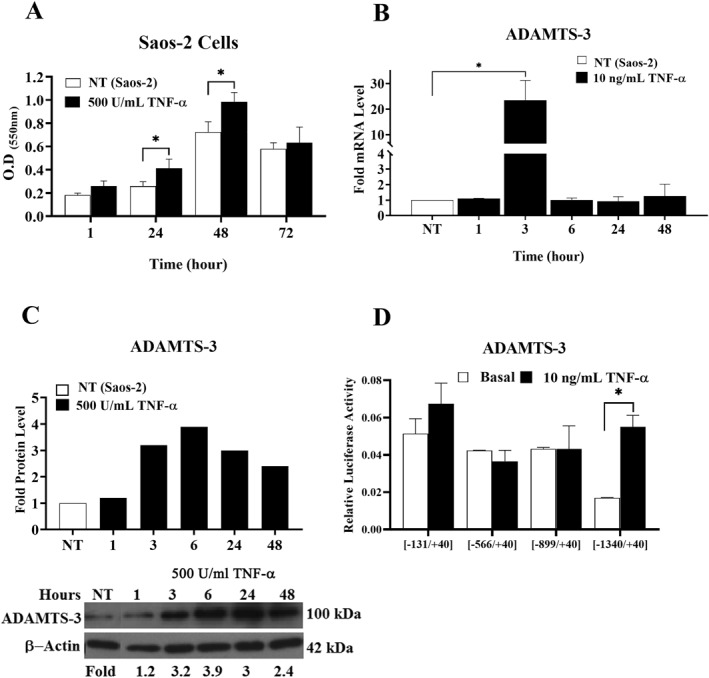
(A) Effects of TNF‐α on Saos‐2 cell proliferation. After serum starvation, Saos‐2 cells were treated with 500 U/mL TNF‐α, and cell viability was assessed using the MTT assay. Data represent mean ± SD from three independent experiments (*p ≤ 0.05). (B) Time course accumulation of ADAMTS‐3 mRNA after TNF‐α administration to Saos‐2 cells. Human β‐2 was used for normalization. The untreated groups were used as controls. The graph represents the mean fold increase in ADAMTS‐3 mRNA levels in TNF‐treated cells compared to untreated cells (*p ≤ 0.05). (C) Effects of TNF‐α stimulation on ADAMTS‐3 protein level. β‐actin was used for normalization. The graph represents the fold increase in ADAMTS‐3 protein level in TNF‐α‐treated cells compared to untreated cells. (D) Effects of TNF‐α on ADAMTS‐3 promoter activity. Saos‐2 cells were transiently transfected with ADAMTS‐3 promoter constructs and then treated with 10 ng/mL of TNF‐α. Untreated groups (basal promoter activities) were used as controls. TNF‐α, tumor necrosis factor‐alpha.

OS frequently promotes local inflammation and activates local immune responses. TNF‐α is a major mediator of the inflammatory response in many diseases, and its role in OS progression has also been demonstrated. Effects of the TNF‐α on bone‐related genes have been investigated in Saos‐2 cells, but the proliferative effect of the mentioned cytokine on Saos‐2 cells hasn't been known yet.^32^, ^33^, ^34^ Here, we first performed an MTT assay to clarify the proliferative effect of TNF‐α on Saos‐2 cells. We determined that TNF‐α induced Saos‐2 cell proliferation in both a rapid (1 h) and a sustained (up to 48 h) manner. Maximum increase was observed at 24 h for 1.6‐fold and at 48 h for 1.4‐fold (*p* ≤ 0.05) (Figure 3A).

### 3.4 TNF‐α transcriptionally activates ADAMTS‐3 expressions in OS model, Saos‐2

Expression patterns of the ADAMTS family members have been studied in normal and tumor tissues. Moreover, the effects of ADAMTSs in certain pathological conditions and across different tumor types have been investigated due to their direct interaction with extracellular matrix components.^35^ ADAMTS‐3 is a well‐expressed metalloproteinase in OS models such as Saos‐2 and MG‐63, as determined in our previous studies.^36^ Here, we investigated the regulatory effects of TNF‐α on ADAMTS‐3 transcription in Saos‐2 cells. It was determined that TNF‐α (10 ng/mL) treatment elevated ADAMTS‐3 mRNA expression by up to 23‐fold for 3 h (Figure 3B). TNF‐α also increased the ADAMTS‐3 protein level starting in the early hours of incubation, with the maximum increase observed at 6 h (3.9‐fold). Inducing effect of TNF‐α on ADAMTS‐3 protein expression was continued up to 48 h (2.4 fold) (Figure 3C).

Next, we investigated the effect of TNF‐α on ADAMTS‐3 promoter activity, given the presence of potential transcription factor binding sites within the ADAMTS‐3 promoter region. ADAMTS‐3 promoter constructs pMET_TS3[−1340/+40], pMET_TS3[−879/+40], pMET_TS3[−576/+40], and pMET_TS3[−131/+40] were transiently transfected into Saos‐2 cells, and TNF‐α was administered after 16 h of transfection. Luciferase and SEAP activities were measured 48 h after transfection in cytokine‐treated or untreated cells. TNF‐α significantly induced pMET_TS3[−1340/+40] promoter activity up to 3.3‐fold. TNF‐α also had a slight inducing effect on pMET_TS3[−131/+40] promoter activity (Figure 3D).

### 3.5 TNF‐α stimulation enhances STAT‐3α and NF‐κB binding to *ADAMTS‐3* promoter in OS

**FIGURE 4:**
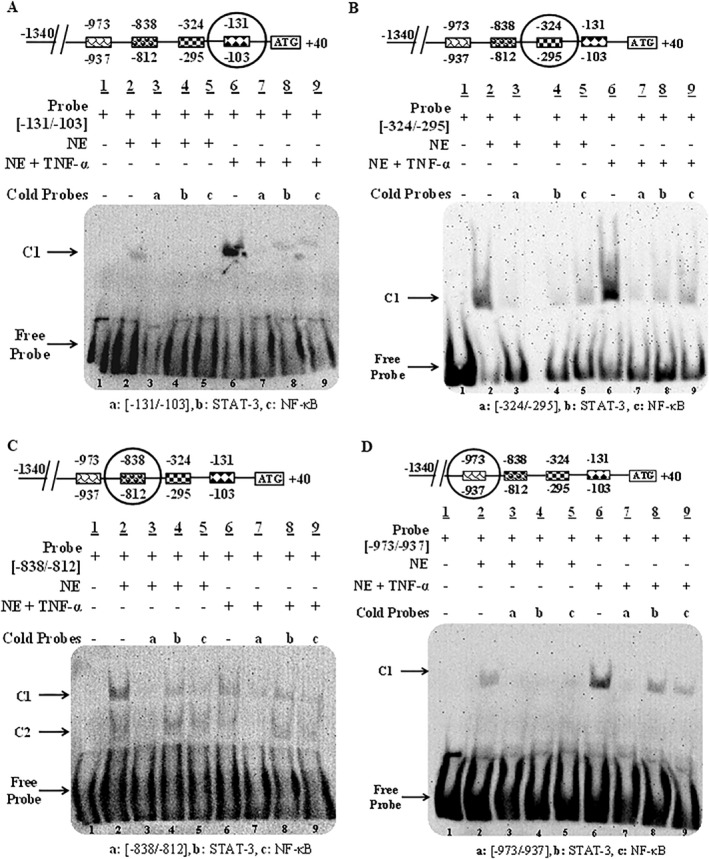
EMSA for in vitro binding analysis of STAT‐3 and NF‐κB transcription factors to ADAMTS‐3 promoter using Saos‐2 nuclear extracts with or without TNF‐α. EMSA with (A) (‐131/‐103) probe (B) (‐324/‐295) probe, (C) (‐838/‐812) probe, (D) (‐973/‐937) probe. C1 and C2 (Complex 1 and 2), NE, nuclear extract; TNF‐α, tumor necrosis factor‐alpha.

**FIGURE 5:**
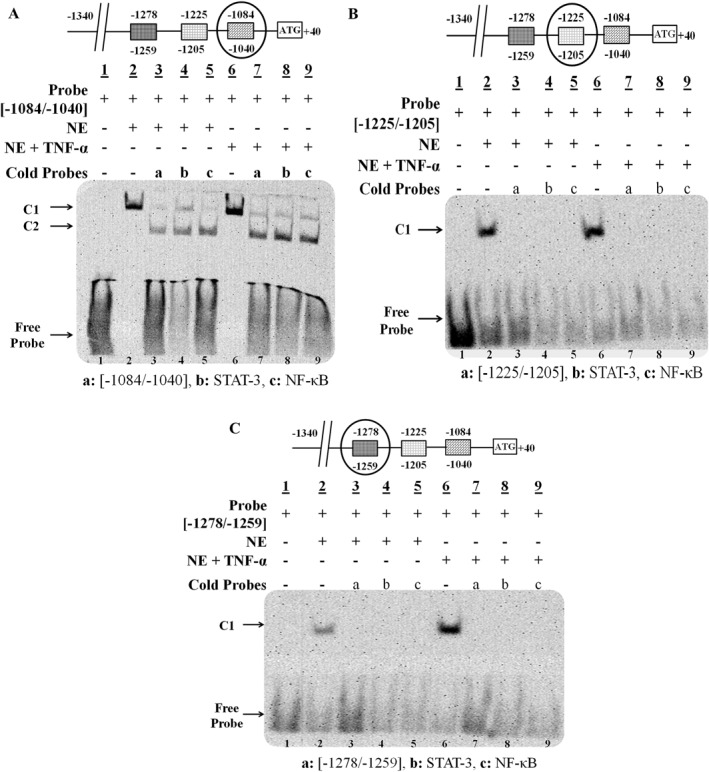
EMSA for in vitro binding analysis of STAT‐3 and NF‐κB transcription factors to ADAMTS‐3 promoter using Saos‐2 nuclear extracts with or without TNF‐α. EMSA with (A) (‐1084/‐1040) probe, (B) (‐1225/‐1205) probe, (C) (‐1278/‐1259) probe. (C1) and (C2) (Complex 1 and 2), NE, nuclear extract; TNF‐α, tumor necrosis factor‐alpha.

We conducted in silico analyses to identify the potential transcription factors that could be involved in the TNF‐α‐mediated upregulation of ADAMTS‐3. MatInspector analysis revealed multiple binding sites for STATs and NF‐κB in the ADAMTS‐3 promoter region that could be functional in the inflammation‐related pathways. EMSA probes were designed for seven different regions in the ADAMTS‐3 promoter corresponding (−131/−103), (−324/−295), (−838/−812), (−973/−937), (−1084/−1040), (−1225/−1205), (−1278/−1259) bp to analyze functional binding of STAT and NF‐κB under TNF‐α stimulation (Table 2). A Complex formation (C1) was observed when biotinylated probes (−131/−103), (−324/−295), (−973/−937), (−1084/−1040), (−1225/−1205), (−1278/−1259) incubated with Saos‐2 nuclear extracts (Figure 4A,B,D; lanes 2 and Figure 5A–C; lanes 2). The specificity of the C1 was confirmed by the addition of the unlabeled probes (Figure 4A,B,D; lanes 3 and Figure 5A–C; lanes 3). In competition assays, the complex (C1) was almost completely abolished either by unlabeled STAT‐3 or NF‐κB consensus oligonucleotides using control Saos‐2 nuclear extracts indicating functional binding of these transcription factors to the corresponding region in the ADAMTS‐3 promoter (Figure 4A,B,D; lanes 4 and 5 and Figure 5A–C; lanes 4 and 5). C1 formation was stronger for all probes when TNF‐α‐treated Saos‐2 nuclear extract was used in binding reactions (Figure 2 and 3, lanes 6), confirming the inducing effect of the TNF‐α on STAT‐3 and NF‐κB proteins that bind these regions.

**TABLE 2:** EMSA primer sequences.

Oligonucleotide name	Oligonucleotide sequence
ADAMTS‐3 [‐131/‐103] F ADAMTS‐3 [‐131/‐103] R	5′GCTCAAATTTCATTTTCATTGAAGCAAAG3′ 5′CTTTGCTTCAATGAAAATGAAATTTGAGC3′
ADAMTS‐3 [‐324/‐295] F ADAMTS‐3 [‐324/‐295] R	5′ATGGTGCTCGAAAAGCCTCGAAAAAGCTGC3′ 5′GCAGCTTTTTCGAGGCTTTTCGAGCACCAT3′
ADAMTS‐3 [‐838/‐812] F ADAMTS‐3 [‐838/‐812] R	5′TCGGACCCTCCCCCTTCCTATAATTAA3′ 5′TTAATTATAGGAAGGGGGAGGGTCCGA3′
ADAMTS‐3 [‐973/‐937] F ADAMTS‐3 [‐973/‐937] R	5′GACTGGTGCCTGGAAGGGAGATCACCGCGTGGTTAAG3′ 5′CTTAACCACGCGGTGATCTCCCTTCCAGGCACCAGTC3
ADAMTS‐3 [‐1084/‐1040] F ADAMTS‐3 [‐1084/‐1040] R	5′TTAGTTTTAAAAGATACCACGTCCTTTCCTTGACTCCACCCGGAT3′ 5′ATCCGGGTGAGTCAAGGAAAGGACGTGGTATCTTTTAAAACTAA3′
ADAMTS‐3 [‐1225/‐1205] F ADAMTS‐3 [‐1225/‐1205] R	5′CTAGGATTCCAGCAAACTCTC3′ 5′GAGAGTTTGCTGGAATCCTAG3′
ADAMTS‐3 [‐1278/‐1259] F ADAMTS‐3 [‐1278/‐1259] R	5′TCTGCTTTCTGGAAAGCACC3′ 5′GGTGCTTTCCAGAAAGCAGA3′
NF‐κB_consensus F NF‐κB_consensus R	5′AGTTGAGGGGACTTTCCCAGGC3′ 5′GCCTGGGAAAGTCCCCTCAACT3′
STAT‐3_consensus F STAT‐3_consensus R	5′GATCCTTCTGGGAATTCCTAGA3′ 5′GATCTAGGAATTCCCAGAAGGATC3′

Two complex formations were detected with (−838/−812) probe using nuclear extracts with or without TNF‐α (Figure 2C, lanes 2 and 6). Complexes weakened in competition assays with an excess amount of unlabeled (−838/−812), STAT‐3 and NF‐κB probes, both control and TNF‐α stimulated extracts (Figure 4C, lanes 3, 4, 5, and 7, 8, 9), indicating STAT‐3 and NF‐κB interaction with the ADAMTS‐3 promoter. However, the intensity of the complex didn't change by TNF‐α treatment. So, it can be concluded that TNF‐α didn't induce binding of STAT‐3 and NF‐κB to this region.

### 3.6 Inhibition of MEK and JNK pathways attenuates the TNF‐α induced ADAMTS‐3 promoter activity

**FIGURE 6:**
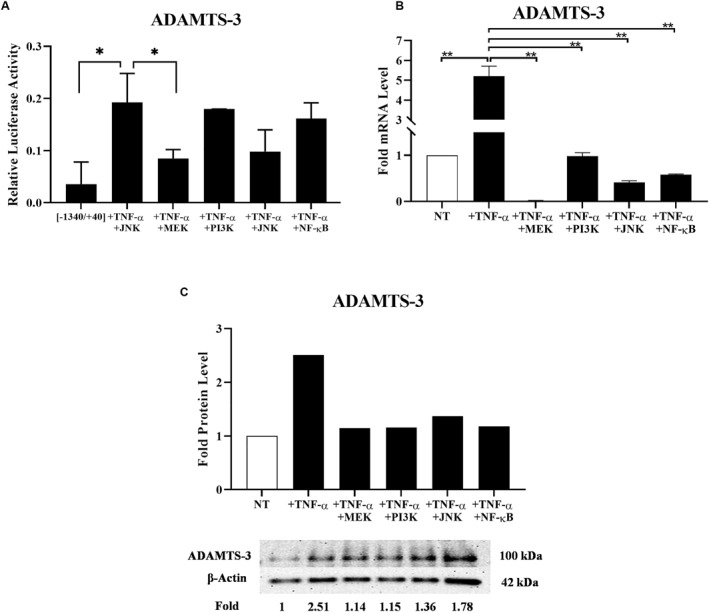
(A) Effect of pathway inhibitors on TNF‐α induced ADAMTS‐3 promoter activity. Luciferase values were normalized to SEAP values. Data represent relative luciferase activity (*p ≤ 0.05). (B) and (C) Effect of pathway inhibitors on TNF‐α‐mediated induction of ADAMTS‐3 mRNA and protein levels. Data represent fold ADAMTS‐3 mRNA and protein values (*p ≤ 0.05, **p ≤ 0.01). TNF‐α, tumor necrosis factor‐alpha.

Next, we investigated the effect of TNF‐α on the ADAMTS‐3 promoter activity because of the presence of potential binding sites of transcription factors that function in TNF‐α signaling in the ADAMTS‐3 promoter region. ADAMTS‐3 promoter constructs pMET_TS3[−1340/+40], pMET_TS3[−879/+40], pMET_TS3[−576/+40], and pMET_TS3[−131/+40] were transiently transfected into Saos‐2 cells, and TNF‐α was administered after 16 h of transfection. Luciferase and SEAP activities were measured 48 h after transfection in control and cytokine‐treated cells. TNF‐α significantly induced pMET_TS3[−1340/+40] promoter activity up to 3.3‐fold. TNF‐α also had a slight inducing effect on pMET_TS3[−131/+40] promoter activity (Figure 6A). To investigate how TNF‐α upregulates ADAMTS‐3 expression, pathway inhibition studies were performed. As it was the most induced fragment by TNF‐α, the pMET_TS3[‐1340/+40] was used in promoter related pathway inhibition studies. Pharmacological inhibitors were introduced into cells transfected with the pMET_TS3[−1340/+40] promoter construct prior to TNF−α stimulation. In cells pretreated with the MEK1/2 (MEK/ERK) inhibitor (PD98059) or the JNK1/2 inhibitor (SP600125), TNF‐α failed to induce ADAMTS‐3 promoter activity, while it maintained its inducing effect on ADAMTS‐3 promoter activity in cells pretreated with the PI3K inhibitor (Wortmannin) or NF‐κβ inhibitor (Bay 11‐7082) (Figure 6B).

### 3.7 TNF‐α induces ADAMTS‐3 mRNA and protein expressions through MEK/PI3K/JNK and NF‐κβ pathways

As we demonstrated before, TNF‐α could increase steady‐state ADAMTS‐3 mRNA and protein levels in a time‐dependent manner. Furthermore, we investigated the signaling pathways involved in the TNF‐α‐mediated induction of ADAMTS‐3 expression. JNK, MEK1/2 (ERKs), p38, and the NF‐κB are the main signal transducers of the TNF‐superfamily.^20^ Therefore, we pretreated Saos‐2 cells with pharmacological inhibitors specific to these pathways before TNF‐α stimulation. TNF‐α‐mediated induction in ADAMTS‐3 mRNA and protein expression was significantly abrogated in cells that were pretreated with MEK1/2 (ERKs), PI3K, JNK, and NF‐κB inhibitors (Figure 6B,C).

## 4 DISCUSSION

OS development and metastasis are complex processes requiring multiple steps and numerous physiological changes. OS activates local immune responses by stimulating local inflammation.^33^ TNF‐α is a key mediator in the inflammatory response.^21^ Critical functions of TNF in bone remodeling have long been known. Also, it is implicated in tumor proliferation and angiogenesis.^22^ Mori and colleagues determined that TNF was required for the tumorigenesis of mesenchymal AX OS cells, and TNF suppression could inhibit OS development by keeping AX cells in an undifferentiated state.^33^ Another group has determined that TNF‐α treatment significantly induced the migration and invasion of U2OS OS cells through inducing MMP‐2 expression.^34^ Kato et al. demonstrated that OS lung metastasis was reduced with anti‐TNF‐α treatment (infliximab).^37^ Our findings indicated that TNF has an increasing effect on the Saos‐2 cell proliferation in accordance with previous studies.

The differential expression of ADAMTS family members in response to inflammatory cytokines was investigated in pathological tissues to understand better the contribution of cytokine‐mediated ADAMTS gene expression changes to disease. For instance, Bevitt and colleagues displayed that TNF‐α stimulation upregulated ADAMTS1,6 and 9 expression in ARPE‐19, so they suggested that ADAMTSs may be involved in the proteolytic modification of the retinal ECM in both normal and pathological conditions.^38^ In another study, it was determined that IL‐1 and OSM synergistically elevated the expression of aggrecan processing enzymes, ADAMTS‐4 and ‐5, in chondrocyte cell lines.^18^ TNF‐α‐mediated induction of ADAMTS‐4 in osteoarthritic chondrocytes was also displayed.^39^ Demircan et al. observed that while IL‐1β could induce ADAMTS‐1, ‐4, ‐5, ‐8, and ‐9 expressions, it did not affect ADAMTS‐1 and ‐8 expressions in OUMS‐27 (human chondrosarcoma) cell line and human chondrocytes.^40^ In a study conducted by Do et al., TNF‐α was found to have a decreasing effect on ADAMTS‐1 protein expression in pre‐adipocyte cells.^19^ IL‐1β‐mediated upregulation of ADAMTS‐1 was demonstrated in human decidual stromal cells.^41^ TNF‐α and IL‐1β elevated ADAMTS‐7 expression in vascular smooth muscle cells.^42^ In our previous studies, we demonstrated that ADAMTS‐2 and ADAMTS‐3 are transcriptionally upregulated by the inflammatory cytokines IL‐6 and IL‐1α in OS. Therefore, it can be concluded that cytokine‐mediated regulation of ADAMTSs exhibits cell‐ and tissue‐specific properties.^14^, ^26^

Here, we performed integrative genomic and transcriptomic characterization of the ADAMTS family in OS, revealing distinct molecular patterns that may contribute to tumor progression. Among the 20 family members analyzed, ADAMTS4, ADAMTS12, ADAMTS16, and ADAMTS17 showed recurrent amplification and high mRNA expression, suggesting potential oncogenic roles. In contrast, other members such as ADAMTS2, ADAMTS6, ADAMTS13, and ADAMTS14 exhibited minimal expression, indicating functional divergence within the family.

Notably, ADAMTS3 emerged as a gene of particular interest. It was consistently overexpressed in OS tissues and cell lines compared with normal osteoblasts. Co‐expression and enrichment analyses demonstrated that ADAMTS3 is functionally associated with biological processes central to OS pathogenesis, including extracellular matrix organization, cell adhesion, and immune response regulation. Positive correlations with MMP2, MMP9, and inflammatory mediators such as IL6, STAT3, and NF‐ĸB suggest that ADAMTS3 operates at the intersection of matrix remodeling and inflammatory signaling. This interaction could facilitate a permissive microenvironment for tumor invasion and metastasis.

The co‐occurrence of ADAMTS3 with inflammatory and EMT‐related gene signatures further supports its potential involvement in tumor–stroma communication and immune modulation. These findings are consistent with reports in other cancers, where dysregulated ADAMTS members influence extracellular matrix degradation, angiogenesis, and the recruitment of immune cells. In OS, where tumor aggressiveness and poor clinical outcomes are tightly linked to matrix dynamics and immune evasion, ADAMTS3 may represent a key mediator connecting these biological processes.

In wet lab experiments, we showed that TNF‐α can induce the significant expression of ADAMTS‐3 mRNA and protein in Saos‐2 cells. This finding indicated that ADAMTS‐3 could have regulatory effects on the OS microenvironment.

Hou and colleagues demonstrated that TNF‐α enhanced chondrosarcoma cell migration by inducing integrin migration through the mitogen‐activated protein kinase (MEK), ERK, and nuclear factor‐κB (NF‐κB) signal transduction pathways.^43^ In the present study, pathway inhibition studies indicated that the MEK, PI3K, JNK, and NF‐κB cascades are responsible for the TNF‐α‐mediated induction of ADAMTS‐3 expression in OS.

In silico analysis of the ADAMTS‐3 promoter indicated multiple STAT and NF‐κB binding sites in the region. Involvement of these transcription factors in TNF‐α signaling has been reported in several other studies. When proinflammatory cytokines are αctivated, JAKs serve as docking sites for signaling molecules, such as STATs.^44^, ^45^ EMSA confirmed that the STAT and NF‐ĸB functionally bind to multiple sites within the ADAMTS‐3 promoter. Moreover, TNF‐α stimulation markedly enhanced the binding activity of these transcription factors to the ADAMTS‐3 promoter, confirming that TNF‐α facilitates transcriptional activation of ADAMTS‐3 through increased promoter occupancy.

Here, we also investigated the regulatory effect of TNF‐α on the ADAMTS‐3 promoter with the transient transfection of truncated ADAMTS‐3 promoter constructs. Our data show that [‐131/+40] and the largest promoter construct [‐1340/+40] were responsive to TNF‐α.

In conclusion, our results highlight a complex regulatory network involving ADAMTS family genes in OS. Specifically, *ADAMTS3* may serve as a *functional driver* of tumor progression through its association with inflammatory and matrix remodeling pathways. It is clear that TNF‐α has an inducing effect on OS cell proliferation. TNF‐α induces ADAMTS‐3 expression at both the mRNA and protein levels, suggesting a potential regulatory role of ADAMTS‐3 in the OS microenvironment. TNF‐α exerts its inducing effect through MEK, PI3K, JNK, and NF‐ĸB signaling pathways. Future studies should validate these findings in larger patient cohorts and investigate the therapeutic potential of targeting ADAMTS3‐mediated signaling in OS under inflammatory conditions. In addition, a key limitation of this study is the absence of in vivo validation to support the in vitro findings. The inclusion of in vivo approaches, such as xenograft or orthotopic OS models, would have strengthened the biological relevance and translational significance of the results.

## AUTHOR CONTRIBUTIONS

The study conception and design, methodology, and formal analysis, writing original draft, and editing were performed by Meltem Alper and Feray Köçkar. Investigation, Methodology, formal analysis, and writing original draft were performed by Ehed Muhammed Aymaz, Feyza Nur Sav and Tuğşen Aydemir. All authors commented on previous versions of the manuscript. All authors read and approved the final manuscript.

## CONFLICT OF INTEREST STATEMENT

The authors declare no conflicts of interest.

## ETHICS STATEMENT

The article does not contain research in which animals or humans were used, so ethical approval is not required for this study.

## Data Availability

The data that support the findings of this study are available from the corresponding author upon reasonable request.
